# Supplementary material for: In Situ Epitaxial Quantum Dot Passivation Enables Highly Efficient and Stable Perovskite Solar Cells
Source: Nanomaterials (Basel). 2025 Jun 24;15(13):978. doi: 10.3390/nano15130978 (PMC12250851; doi:10.3390/nano15130978)
Supplement: Supplementary file 1 [file nanomaterials-15-00978-s001.zip › nanomaterials-3659770-supplementary.pdf]

Supporting information:

In Situ Epitaxial Quantum Dot Passivation Enables Highly Efficient and Stable Perovskite Solar Cells

Yahya A. Alzahrani[1], Raghad M. Alqahtani[2], Raghad A. Alqarni[2], Jenan R. Alnakhli[2], Shahad A. Anezi[2], Ibtisam S. Almalki[1], Ghazal S. Yafi[3], Sultan M. Alenzi[1], Abdulaziz Aljuwayr[1], Abdulmalik M. Alessa[1], Huda Alkhaldi[2*], Anwar Q. Alanazi[1], Masaud Almalki[1], Masfer H. Alkahtani[1*]

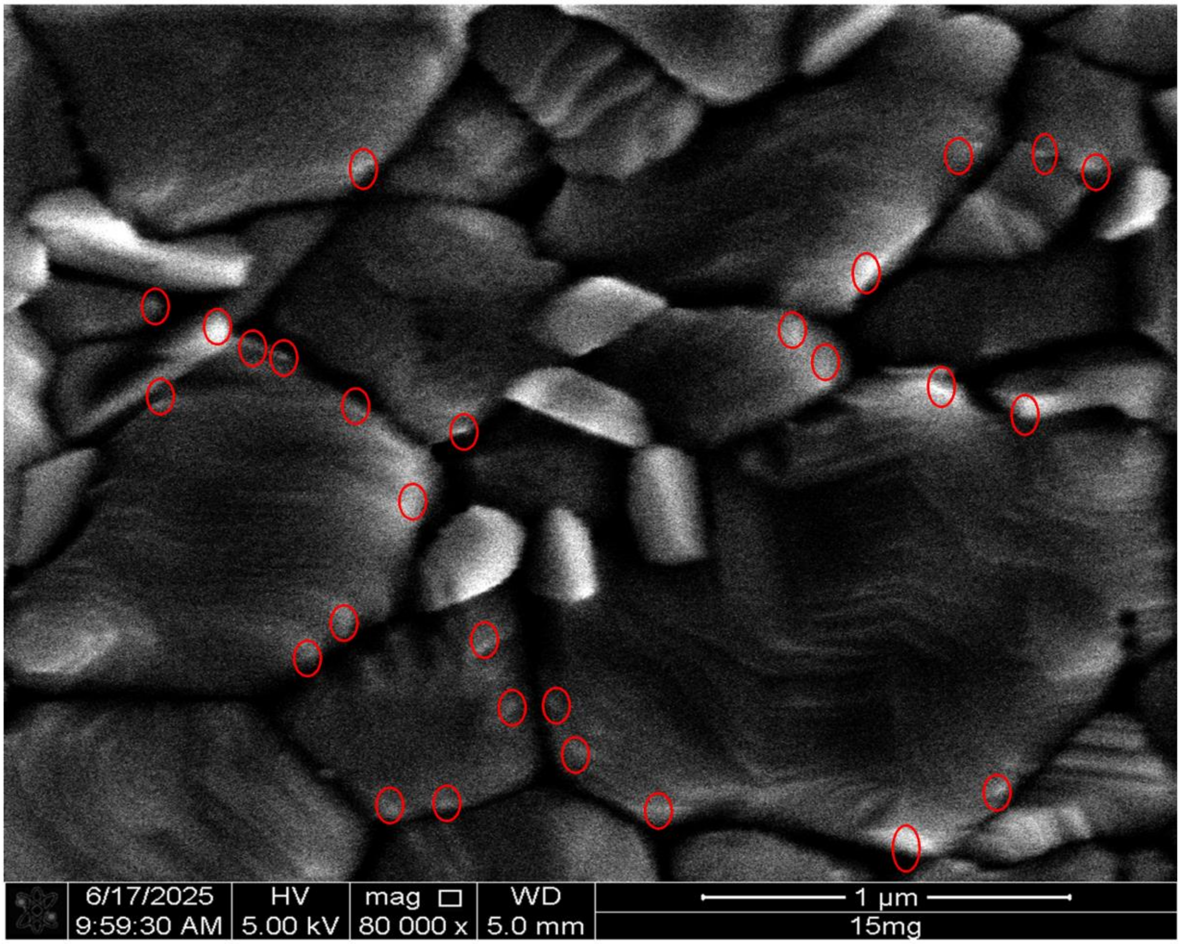

Figure S1. High-magnification SEM images of perovskite films treated with the optimized PQD concentration (15 mg/mL). The images reveal that PQDs are predominantly localized at the grain boundaries, indicating their role in passivating interfacial defects and improving film morphology. This distribution supports enhanced structural coherence and reduced non-radiative recombination at grain interfaces.

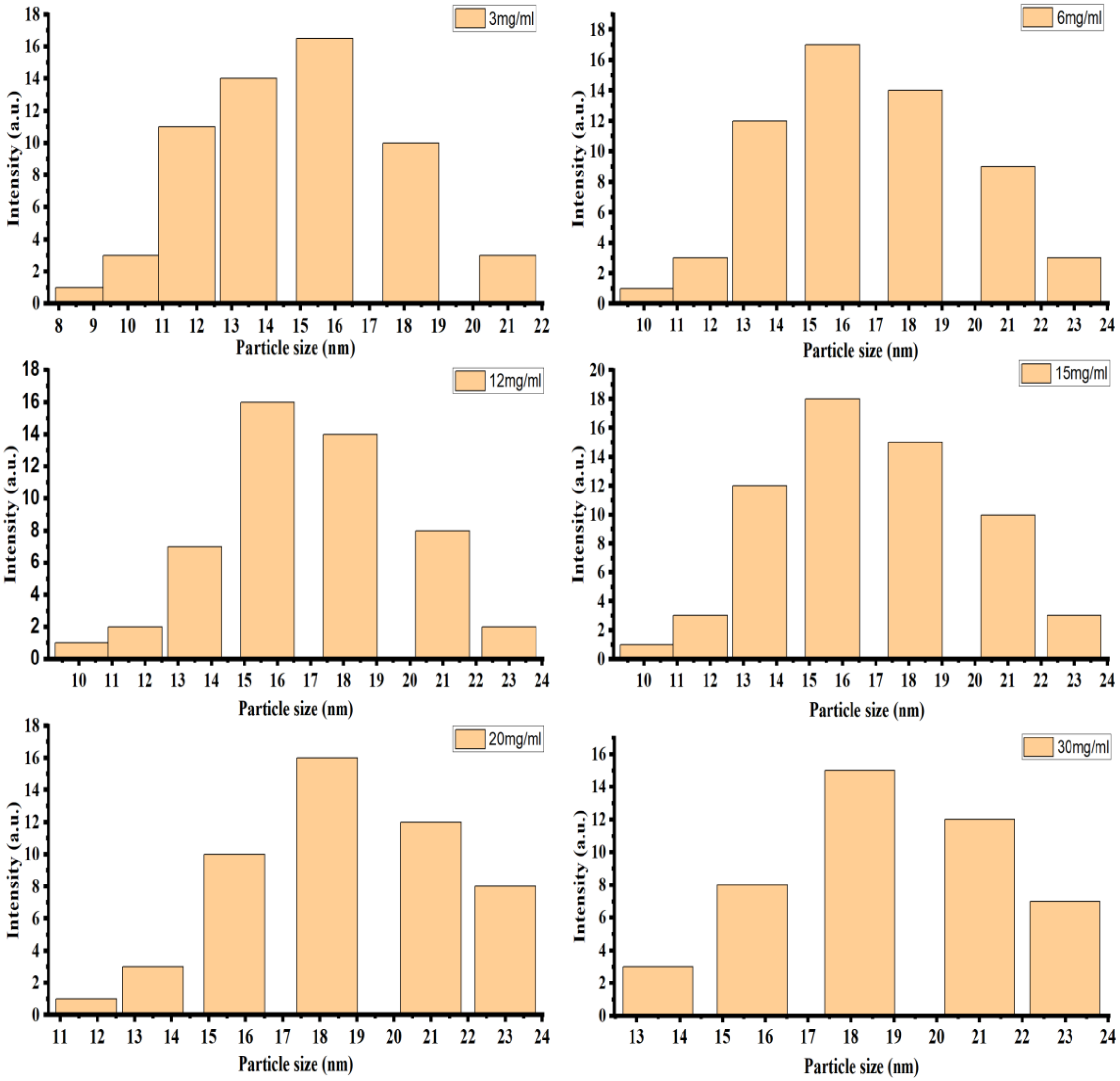

Figure S2: Dynamic light scattering (DLS) analysis of the PQD dispersion at different concentrations prior to incorporation into the perovskite film. The results show a narrow particle size distribution and no significant aggregation, confirming good colloidal stability and ensuring uniform PQD integration during the passivation process.
